# Under utilization of long-lasting insecticidal nets (LLINs) is challenging malaria elimination program in Ethiopia: a systematic review and meta-analysis

**DOI:** 10.1186/s12889-024-18344-w

**Published:** 2024-03-15

**Authors:** Fekade Demeke Bayou, Natnael Kebede, Yawkal Tsega, Shambel Nigussie, Temesgen Dessalegn Legassu, Amare Muche, Ayana Alebachew Muluneh, Fanos Yeshanew Ayele

**Affiliations:** 1 Department of Epidemiology and Biostatistics, School of Public Health, College of Medicine and Health Sciences, Wollo University, Dessie, Ethiopia; 2 Department of Health Promotion, School of Public Health, College of Medicine and Health Sciences, Wollo University, Dessie City, Ethiopia; 3 Department of Health System and Project Management, School of Public Health, College of Medicine and Health Sciences, Wollo University, Dessie City, Ethiopia; 4 Department of Clinical Pharmacy, School of Pharmacy, College of Health and Medical Science, Haramaya University, Harar, Ethiopia; 5 Department of Midwifery, College of Medicine and Health Sciences, Jigjiga University, Jigjiga, Ethiopia; 6 Department of Health Informatics, College of Medicine and Health Sciences, Wollo University, Dessie, Ethiopia; 7 Department of Public Health Nutrition, College of Medicine and Health Sciences, Wollo University, Dessie City, Ethiopia

**Keywords:** LLIN, Utilization, Systematic review, Meta-analysis, Ethiopia

## Abstract

**Background:**

Malaria is one of the most common causes of morbidity and mortality in developing countries including Ethiopia. Mass distribution of insecticide-treated nets and indoor residual spray for high malaria risk groups are the major prevention measures in different countries. Achievement of the malaria elimination plan is highly determined by the level of effective utilization of intervention measures. However, there is scarce information showing the national level of insecticide-treated nets utilization.

**Objective:**

To estimate the pooled prevalence of insecticide-treated nets utilization in Ethiopia, 2023.

**Method:**

A Systematic Review and Meta-analysis employed to assess the utilization of long-lasting insecticidal nets in Ethiopia. Published articles were searched from Google Scholar, PubMed, Web Sciences, CINAHIL, EMBASE, and Scopus. The collected articles were screened for data extraction and further analysis using preferred reporting items for systematic review and meta-analysis (PRISMA) flow chart. The quality of each study was assessed using the Jonna Briggs Institute (JBI) checklist. The data were extracted using Microsoft Excel and exported to STATA version 17.0 for analysis. The overall pooled prevalence of long-lasting insecticidal nets utilization was determined using a random effects model.

**Result:**

Out of 1657 articles reviewed, only 21 of them were eligible for final analysis. All of the included studies were used to estimate the pooled prevalence of long-lasting insecticidal net utilization. The point prevalence of LLIN utilization ranged from 14.23 to 91.9%. The Meta-analysis estimated that the overall pooled prevalence of insecticidal nets utilization among all study participants in Ethiopia was 56.26% (95%CI: 44.04–68.48%). Subgroup analysis revealed that insecticidal nets utilization was relatively highest in the Amhara region [63.0, 95%CI (37.0–89.0%)] and during 2020–2023 [61, 95% CI (53.0–69.0%)].

**Conclusion:**

Long-lasting insecticidal nets utilization in Ethiopia is lower than the national target plan. Hence, it needs extra follow-up and intervention to enhance its utilization.

**Supplementary Information:**

The online version contains supplementary material available at 10.1186/s12889-024-18344-w.

## Background

Malaria is one of the commonest causes of morbidity and mortality in developing countries, mostly in poor tropical and subtropical areas of the world [1]. In 2021, nearly half of the world’s population was at risk of malaria, and nearly 619, 000 died. The WHO African Region carries a disproportionately high share of the global malaria burden [2]. Globally, there was a tremendous reduction in morbidity and mortality of malaria; for instance, malaria deaths reduced steadily over the period 2000–2019, from 896,000 in 2000 to 562,000 in 2015 and 558,000 in 2019 [3]. Moreover, evidence showed that malaria incidence rates had decreased by 37% globally and mortality rates by 60% from 2000 to 2015 [4]. However, recently, the number of malaria cases has increased from 245 million (2020) to 247 million (2021). Between 2019 and 2021, an estimated additional 13.4 million cases and 63, 000 deaths were attributed to disruptions during the COVID-19 pandemic [3, 5]. There is a commitment to reduce malaria case incidence and mortality rate by at least 75% by 2025 and 90% by 2030 [6]. As part of this ambitious plan, ITNs were supplied globally in a wider range: for instance, in 2021 alone, a total of 128 million ITNs were distributed [5].

Ethiopia is one of the malaria-endemic countries in Africa suffering a pooled prevalence of 13.61% among adults [7], 22.03% among under-five children [8], and 12.72% among pregnant women [9]. In 2020, the annual reported number of malaria cases was 1,848,231 with 173 deaths [10]. In Ethiopia, since 2008, more than 47 million long-lasting insecticide-treated bed nets have been procured and distributed across the country through the U.S. President’s Malaria Initiative (PMI) [11]. The government of Ethiopia in collaboration with supporting organizations and initiatives is striding to end malaria by 2030. In its malaria elimination program, Ethiopia has aimed to reduce malaria morbidity and mortality by 50% (from the baseline of 2020), to achieve zero indigenous malaria in districts with annual parasite incidence of less than 10, and to prevent the reintroduction of malaria in districts reporting zero indigenous malaria cases. To realize its goal, the country specifies vector control as a prior intervention through improving appropriate LLIN utilization, indoor residual spraying (IRS), and larval source management [12, 13]. As a result, the mass distribution of ITNs and IRS for high malaria risk groups are the major vector control measures under implementation [14]. In fact, beyond wide coverage, the achievement of the proposed target plan is highly determined by the level of effective utilization of intervention measures among the target population. The aggregation of multiple individual studies in a systematic and scientific approach is known to provide more precise and better-quality information for decision-making. However, there is scarce information showing the national level of ITN utilization. The main research question answered by this study was: “What is the pooled prevalence of long-lasting insecticidal nets utilization among the population in Ethiopia?” Hence, this systematic review and meta-analysis aimed at determining the pooled prevalence of ITN utilization in Ethiopia.

### Methods and materials

The protocol for this Systematic Review and Meta-Analysis was registered at PROSPERO with registration number CRD42023412232. We have amended some parts of the protocol during actual study. The overall study processes were accomplished from March to May 2023. We also adhered to the Preferred Reporting Items for Systematic Review and Meta-Analysis checklist.

### Information source and search strategy

This systematic review and Meta-analysis identified individual studies conducted on the utilization of LLINs to synthesize a pooled summary of such evidence. The presence of previously published work on similar topics was checked to avoid duplication. Search engines including Google Scholar, PubMed, Web Sciences, CINAHIL, EMBASE, and Scopus were accessed to search articles. In addition, national and institutional repositories were retrieved to accommodate gray literature. Four research experts (FDB, YT, SN, TDL) extensively searched articles from March 28 to April 20, 2023. Keywords like “utilization”, “use”, “Practice”, “adherence”, “prevalence”, “magnitude”, “level”, “insecticide-treated nets”, “Long lasting insecticidal nets”, “LLINs”, “bed nets”, “mosquito nets”, “net”, “Ethiopia”..etc. were conjoined by Boolean operators (AND, OR) to form Medical Subject Heading (MeSH) terms during the searching processes. Using the above keywords and Boolean operators the search strategy formed using PMC Advanced Search Builder was [((((prevalence [Title] OR magnitude [Title] OR level [Title])) AND (utilization [Abstract] OR use [Abstract] OR adherence [Abstract] OR practice [Abstract])) AND (“long-lasting insecticidal nets” OR “insecticide-treated nets” OR “bed nets” OR “mosquito nets” OR Net*)) AND Ethiopia]. Endnote version 20 was used to manage searched literature and to remove duplicated articles.

### Eligibility criteria

#### Inclusion criteria

Study type (design) and setting: cross-sectional studies conducted from January 2013–2023 (GC), in Ethiopia regardless of the study settings (facility-based or community-based) were included. Population or study participants: Studies conducted among any segment of the population including the general population, pregnant women, or children were considered in this SRMA. Language: articles written/published in the English language, which reported the prevalence of ITNs utilization were included in this review.

#### Exclusion criteria

Studies which has poor quality, lack basic information on sample size, outcome (ITN utilization), or incomplete texts (abstracts or inaccessible full texts) were excluded in this Systematic Review and Meta-Analysis.

### Outcome measurement

The outcome variable of this review was the prevalence of LLIN utilization in Ethiopia. It was measured by observation and/or self-reported by the study participants. The pooled prevalence was estimated from the number of individuals who participated and utilized LLIN in each study using the “meta prop” command of STATA.

### Quality assessment and screening strategy

**Fig. 1:**
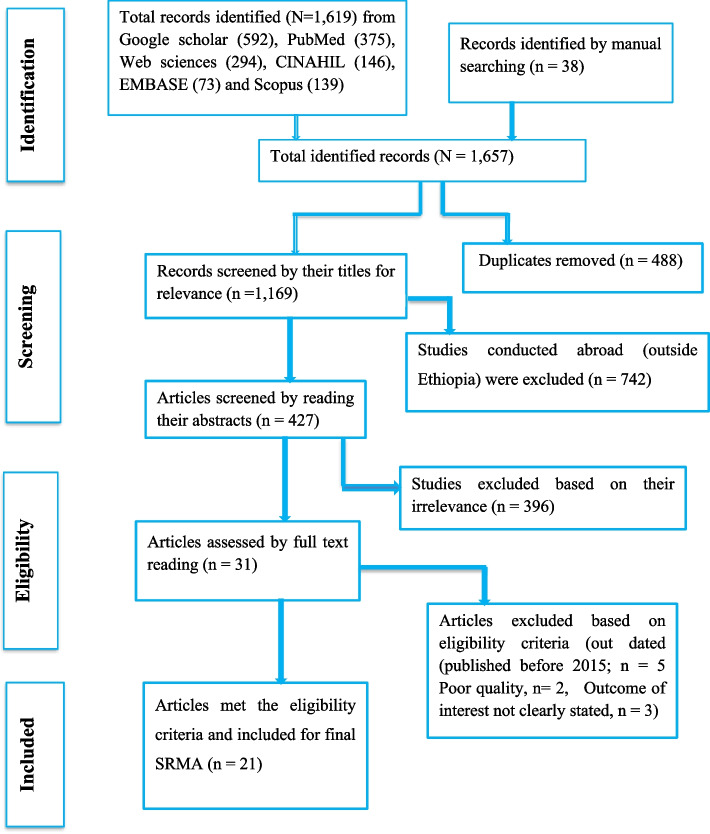
Preferred Reporting Items for Systematic Review and Meta-Analysis (PRISMA) flow chart

The searched articles were compiled and duplicates were removed using Endnote X7. Articles were also screened based on their titles, abstracts, and full-text readings. The quality of each study was assessed using the Jonna Briggs Institute (JBI) checklist. Studies with poor quality, with a score of less than 50% according to the JBI checklist, were excluded from the analysis. The screening and quality assessment process was undertaken by two researchers (FDB and YT) independently, and a third person (SN) was involved in case of disagreements between the results of the assigned investigators. The screening process was done by the preferred reporting items for systematic review and meta-analysis (PRISMA) flow chart (Fig. 1).

### Data extraction strategy

Articles that met the inclusion criteria (cross-sectional studies that reported the prevalence of LLIN utilization in Ethiopia and scored 50% and above in JBI quality assessment criteria) were selected for data extraction. Then the data were extracted using a data abstraction checklist. Year of publication, year in which the study was conducted, name of the first author, study area (region), study design, residence, sample size, response rate of the survey, sampling technique, utilization status (of insecticide-treated nets), and study quality score were included in the data abstraction checklist. Two persons (FYA and FDB) extracted the data and disagreements were addressed with the assistance of a third person (TDL).

### Data analysis and synthesis

The data extracted on a Microsoft Excel spreadsheet was exported to STATA software version 17 for analysis. The characteristics of the included studies were described using descriptive statistics. The presence of methodological and statistical heterogeneities among the studies was assessed. Graphically, a Funnel plot was drawn to show if there was any publication bias. Egger’s statistical test was used to check the statistical significance of publication bias. Moreover, the Trim and fill technique was used to see the number of studies needed to adjust publication bias. The pooled prevalence and standard errors were generated using the data extracted on the number of LLIN users and total participants in each included study. Since the data were highly heterogeneous, a random effects model was fitted to measure the pooled effect size with 95% confidence interval. Subgroup analysis was done based on potential categorical variables. The individual and pooled effect sizes were presented by using forest plots.

## Result

### Characteristics of included studies

More than 1,650 articles were accessed from different electronic databases. Out of which, 21 [15] individual studies met the eligibility criteria of this systematic review and meta-analysis. All of the studies were cross-sectional studies, two studies were surveys conducted among the cohort population [16] and as part of a community trial [17]. Almost all (20 out of 21) articles were published in peer-reviewed journals and their response rate ranged from 90.3–100%. All except one [18], national survey, of individual studies were conducted at the regional level. One-third (seven) of the studies were done in the South Nation, Nationalities and Peoples’ (SNNP) region followed by the Oromia region (five studies), Amhara region (four studies), Tigray region (three studies), Gambella region (one study). Regarding the study populations, more than half (11 out of 21) of the studies assessed utilization of LLINs among the general population (community level) while the remaining were conducted among pregnant women (six studies) [15, 19–23], children (three studies) [17, 24, 25] and armies (one study) [26]. A total of 34,652 study participants were included in this systematic review and meta-analysis with the minimum and maximum sample sizes of 268 [27] and 17,142 [16] respectively. The majority of the studies (13 out of 21) were published between 2015 and 2020 (Table 1).

**Table 1:** Characteristics of included studies, SRMA, Ethiopia, 2023

Authors	Region	Study year	Study population	Study Design	Sample Size	Number of Outcomes	Response Rate	Publication Year	JBI Score (%)
Solomon et al.	Oromia	2017	Community	Cross sectional	17,142	2439	100	2019	85
Zerdo et al.	SNNP	2019	Children	Cross sectional	2304	914	98.1	2020	75
Tassew et al.	SNNP	2014	Community	Cross sectional	540	388	94.24	2017	83
Mekuria et al.	Oromia	2022	Community	Community-based CS	550	384	96.7	2022	68
Nadew et al.	SNNP	2021	Pregnant Mother	Community based CS	459	248	94.77	2022	78
Seyoum et al.	SNNP	2015	Community	Community based CS	833	520	97.8	2017	81
Mengistie K	Amhara	2017	Pregnant Mother	Community based CS	422	140	98.8	2021	59
Tesfaye et al.	Oromia	2017	Pregnant Mother	Community based CS	444	169	95.5	2022	68
Alemu et al.	Amhara	2017	Community	Community based CS	268	239	97.00	2018	89
Birhane et al.	Tigray	2016	Armies	cross-sectional study	326	95	100	2019	76
Yirsaw et al.	Amhara	2020	Community	Community-based CS	724	409	100	2021	84
Yitayew et al.	Amhara	2018	Pregnant Mother	Hospital based CS	226	160	100	2018	64
Berkessa et al.	Oromia	2013	Community	Community based CS	636	460	90.3	2016	76
Welyou et al.	SNNP	2020	Children	Cross-sectional	591	344	100	2023	66
Teklit et al.	Tigray	2017	Pregnant Mother	Community-based CS	550	347	100	2020	73
Amanuel et al.	SNNP	2016	Pregnant Mother	Community based CS	630	445	97.5	2018	87
Asnakech et al.	Oromia	2016	Community	community based CS	422	310	100	2016	84
Amha et al.	SNNP	2016	Under 5 Children	Community based CS	413	137	96.35	2018	82
Aklilu & Worku	Gambella	2015	Community	Community based CS	845	361	100	2016	79
Wondatir et al.	National	2017	Community	Community based CS	5660	2354	96.3	2019	77
Girmay et al.	Tigray	2013	Community	Community based CS	667	445	97.3	2015	82

*CS* Cross Sectional, *SNNNP* South Nation Nationalities and Peoples region, *JBI* Jonna Briggs Institute

### Pooled prevalence of LLIN utilization

**Fig. 2:**
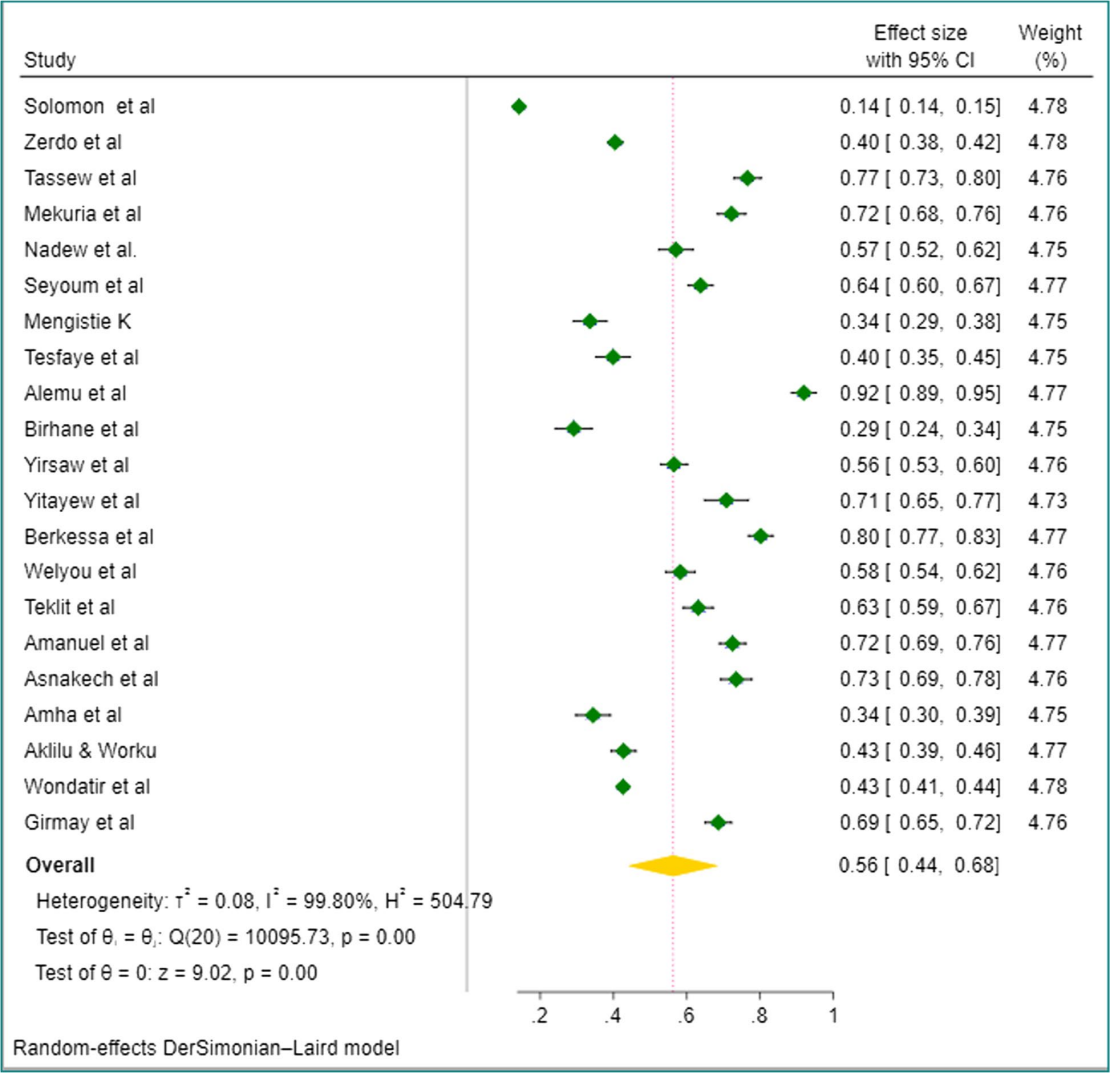
Forest plot of LLIN utilization in Ethiopia: Systematic Review and Meta-Analysis, 2023

All of the included studies were used to estimate the pooled prevalence of long-lasting insecticidal net utilization. The point prevalence of LLIN utilization was ranged from 14.23% [16] to 91.9% [27]. There was a higher level of statistical heterogeneity among individual studies regarding their effect size (I^2^ = 99.80%, *p* < 0.001). We have also noticed the presence of methodological heterogeneity due to the variability of individual studies regarding the study participants, study period, and area. Hence, the random effects model was fitted to accommodate the identified heterogeneity. Moreover, subgroup analysis was employed based on selected categorical variables. The estimated overall LLIN utilization among all study participants in Ethiopia was 56.0% (95%CI: 44.0–68.0%) (Fig. 2).

### Publication Bias assessment

**Fig. 3:**
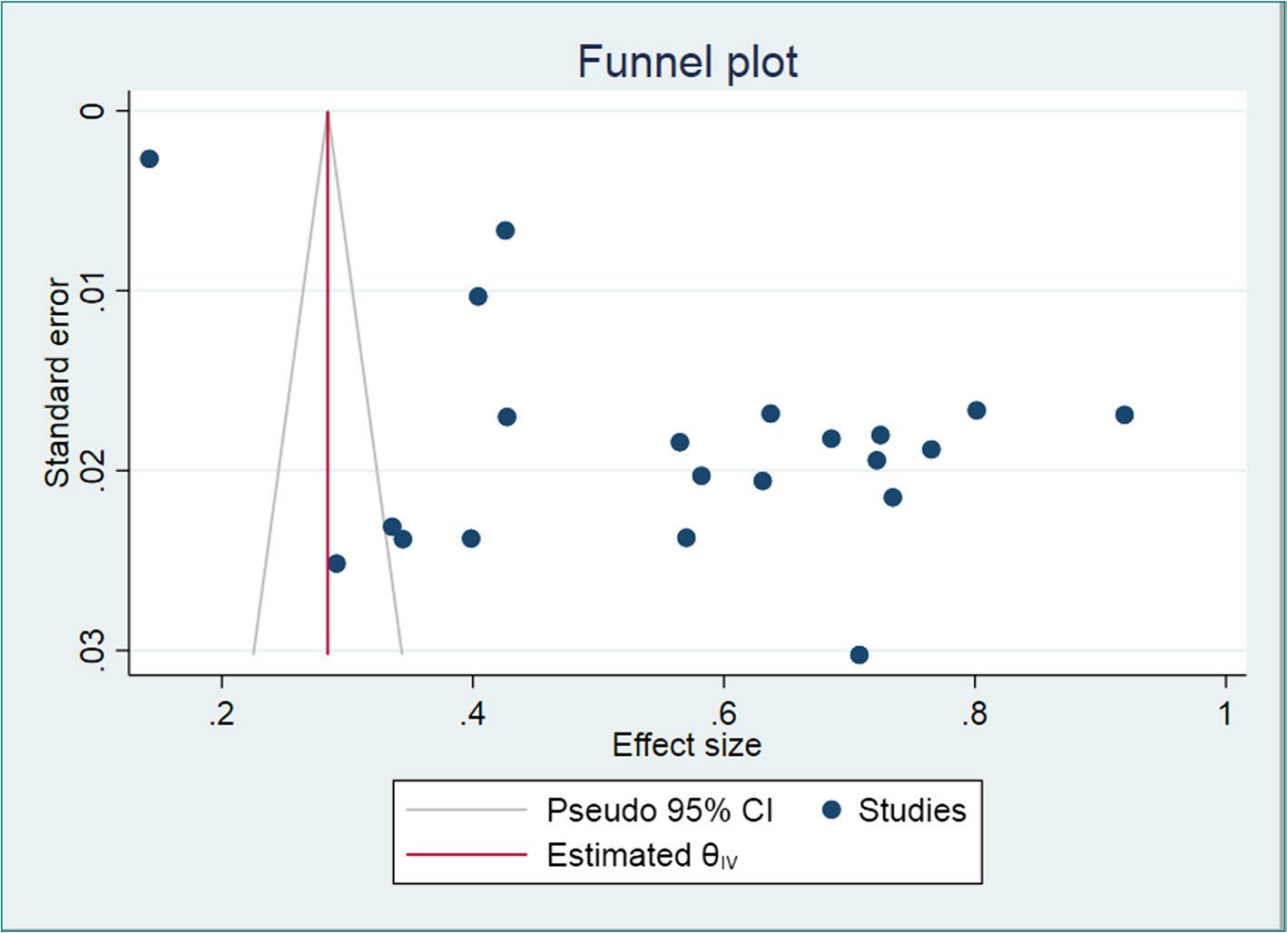
Funnel plot used to assess publication bias

The funnel plot as a graphical diagnosis of publication bias indicates that studies were not symmetrically distributed along the central axis, which illustrates the presence of publication bias, due to the reporting of findings showing higher prevalence or underreporting of studies with lower magnitude (Fig. 3). Objectively, we diagnosed the presence of small study effect using egger’s test (*p* < 0.01).

## Subgroup analysis

### LLIN utilization by regions

**Fig. 4:**
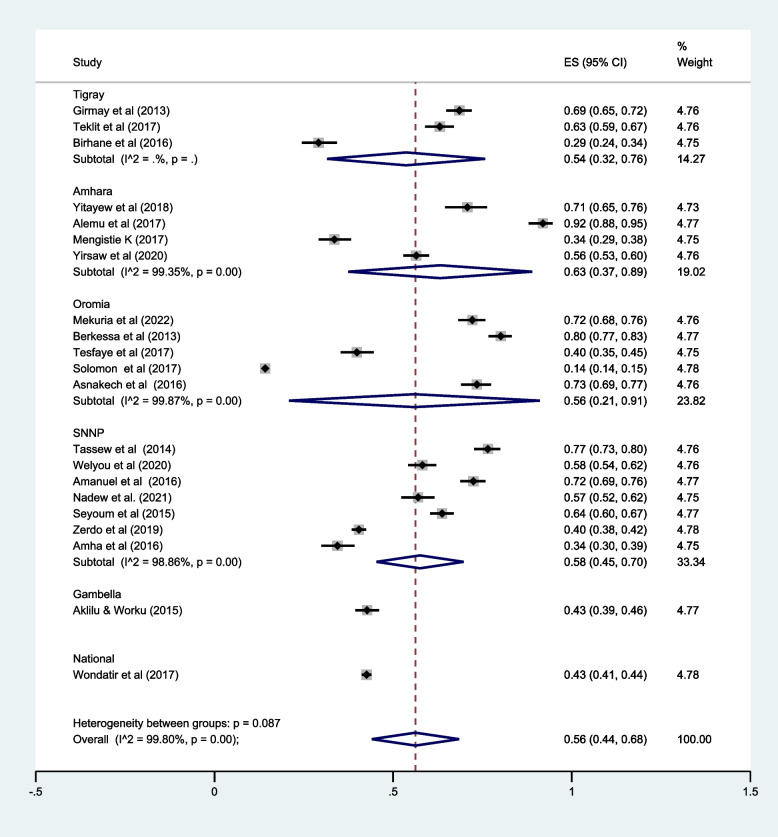
Subgroup analysis of LLIN utilization by Ethiopian regions

This review included studies conducted in five regions including the Tigray region, Amhara region, Oromia, Gambella, and SNNP regions. Individual studies were nearly equally distributed by regions except the Gambella region (a single study was conducted). Based on subgroup analysis, the pooled prevalence of LLIN utilization was highest in the Amhara region [63.0, 95%CI (37.0–89.0%)] followed by the SNNP region [58.0, 95% CI (45.0–70.0%)] (Fig. 4).

### LLIN utilization by population category

**Fig. 5:**
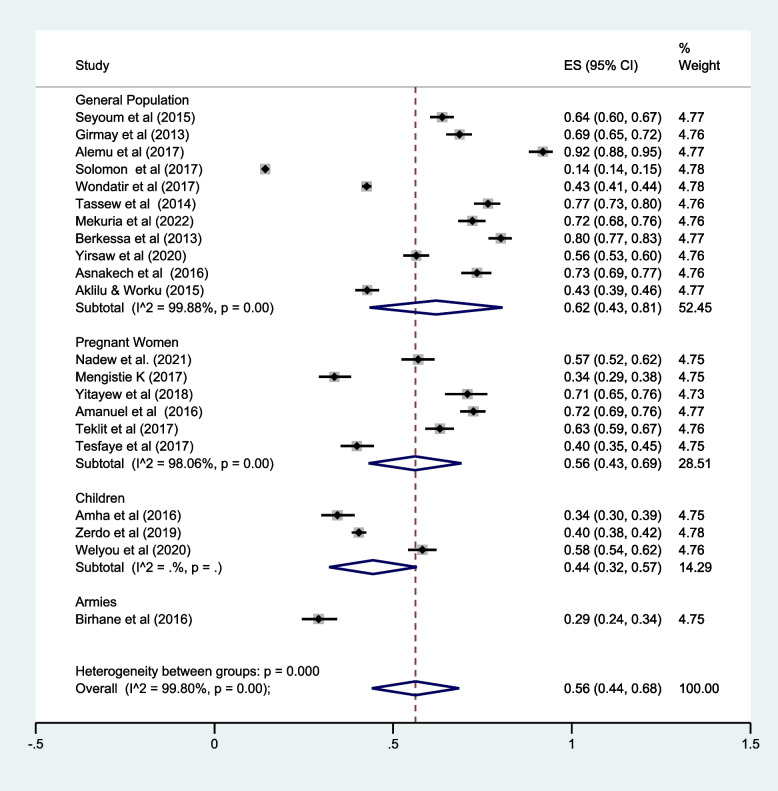
Subgroup analysis of LLIN utilization by population category, SRMA, Ethiopia, 2023

The study participants were children, pregnant women, Armies, and the general population. More than half of the studies (11 out of 21) assessed LLIN among the general population (community-level studies). The overall pooled prevalence of long-lasting insecticidal nets utilization among the general population was 62.0% (95% CI: 43.0–81.0%) (Fig. 5).

### LLIN utilization by study year

**Fig. 6:**
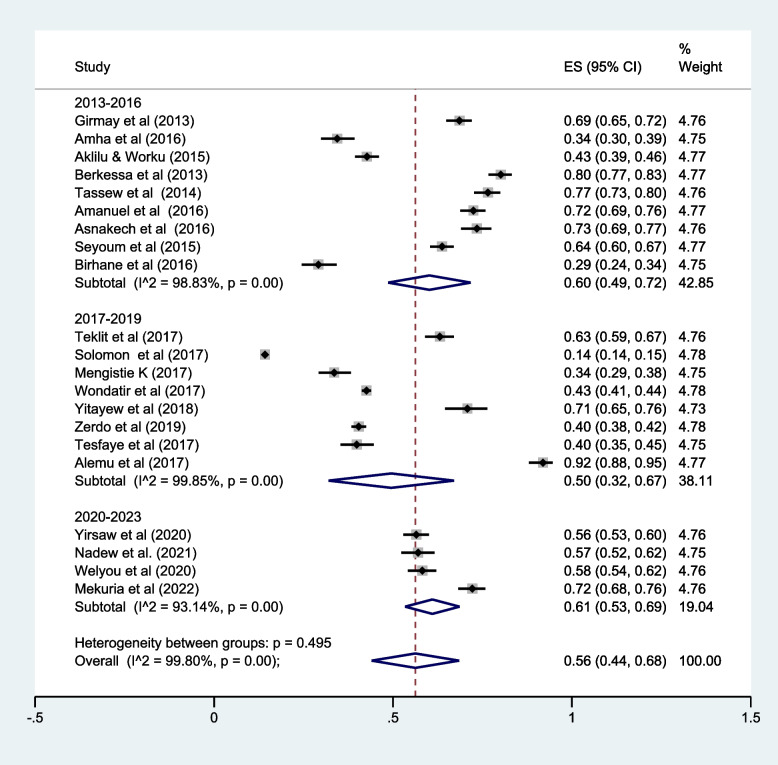
Subgroup analysis by the study year

Regarding the study period, nine studies were done during 2013–2016, eight studies during 2017–2019, and the remaining four studies were done during 2020–2023. Subgroup analysis of LLIN utilization was done by three categories of the study periods (2013–2016, 2017–2019, and 2020–2023). Although there were no statistically significant differences, the result revealed that the pooled prevalence of LLIN utilization was relatively highest during 2020–2023 [61, 95% CI (53.0–69.0%). The lowest level of LLIN utilization was among the studies conducted between 2017 and 2019 [50.0, 95% CI (32.0–67.0%)] (Fig. 6).

## Discussion

In this study, designed to establish the pooled prevalence of long-lasting insecticidal nets (LLIN) utilization among the general population in Ethiopia, we found that 56.0% of the general population in Ethiopia utilized LLINs, with a 95% confidence interval of 44.0 to 68.0%. Since prior data were fragmented and often focused on specific regions or populations, this study offers a needed national-level estimate of LLIN utilization in Ethiopia. This baseline figure allows for better tracking of progress toward malaria control goals and identifying areas where interventions are most needed. The study finding suggests that the current malaria eradication program, which prioritizes LLIN distribution and access, is having a positive impact. The current finding showed that the level of LLIN utilization in Ethiopia lagged behind the national malaria eradication plan, which aimed at increasing the coverage (with one type) of globally recommended vector control intervention by 100% among the population at risk of malaria. Again, this may challenge the country’s malaria elimination plan to end indigenous malaria in the country by 2030 and to see malaria-free Ethiopia [12, 13].

The pooled prevalence of LLIN utilization found in this study was almost consistent with the figure reported by a systematic review and meta-analysis of studies in Sub-Saharan Africa, which found the overall usage of insecticidal nets was 58.3% [28]. The observed concordance might be due to similarities of the study population, which means the majority of Sub-Sahara African populations share similar socio-economic characteristics which are potential determinants of health-seeking behavior. Another reason might be the similarity of the two studies in terms of study periods, studies published between 2015 and 2020 were included in the above study (conducted in Sub-Saharan Africa). The level of LLIN utilization found by the current study was also in line with findings from studies conducted in Côte d’Ivoire (65.4%) [29], Ekiti State, Nigeria (67.6%) [30], Central India (59.4%) [31]. Moreover, our finding on the level of LLIN utilization was comparable with the finding (51%) from a similar study conducted among pregnant women in Ethiopia [32].

However, the current figure is higher as compared with the finding reported by a meta-analysis which compiled studies from Africa, Asia, and South America (41.2%) [33]. The observed discrepancy might be due to study time variation. The above study included studies that were published since 2008, while the current study included studies published since 2015. Populations at different times may have different levels of awareness, access, and utilization of health services. Moreover, individual studies conducted in Cameron (14.1%) [34], Ghana (41.7%) [35], and Uganda (39.5%) [36], reported lower figures on LLIN utilization as compared with the figure found by the current review (56.0%). The observed difference might be due to variations in the studies regarding coverage of LLIN, operational definitions used to measure the outcome variable, and LLIN ownership status of study populations. For instance, the study undertaken in Cameron assessed universal LLIN utilization in the household, which was stricter than the operational definitions used by the studies included in this review.

On the other hand, the level of LLIN utilization found by the current review is lower than the finding from the studies conducted in Uganda (91.1%) [37], Cameroon (72.6%), and Sierra Leone (77.1%) [29], Igabi, Kaduna, Nigeria (70.0%) [38]. The possible justification for these discrepancies might be differences in sample size (a smaller sample was used by the above studies), ownership of LLIN, and time of the study. For example, the study conducted in Uganda was done immediately (6 months) after the mass distribution of LLIN almost, 99% of participants owned at least two nets during the study [37]. The other reason might be due to variation in study populations, i.e. our review assessed LLIN utilization among any population (including the general community, pregnant women, children, and other groups) regardless of their residence (Urban and rural). However, the above studies included women of childbearing age [38] and Urban residents [37].

### Strength and limitation

In this systematic review and meta-analysis, individual studies were summarized to give scientific evidence about the topic for planners and decision-makers at regional and national levels. However, this review might have the following limitations: since individual studies were not equally employed across all regions, caution should be considered while concluding this finding. Higher heterogeneity was also observed among individual findings, which might be due to variations in the measurement method used. Subgroup analysis was used to manage the observed heterogeneity, however, still couldn’t control the heterogeneity.

## Conclusion

The finding of this Systematic Review and Meta-Analysis indicated that long-lasting insecticidal net utilization in Ethiopia is below the national target plan to eradicate malaria. Although not statistically significant, higher prevalence of LLIN utilization was seen in the Amhara region, during 2020–2023, and among the general population (than pregnant women, children, and armies). This finding implies that the malaria eradication program through the application of LLIN utilization is a potential challenge. The level of LLIN utilization was also against the current figure of LLIN coverage in the country. The finding conveys that increasing LLIN coverage alone couldn’t achieve the desired outcome. In general, this is an alert for health planners and decision-makers at different levels of malaria eradication programs to give special attention to the follow-up regarding utilization. Hence, we would like to remind the need for integrated efforts to maximize the level of insecticide-treated nets utilization among malaria risk groups to achieve a malaria eradication program in the country.

### Supplementary Information

**Supplementary Material 1.**

**Supplementary Material 2.**

## Data Availability

All data are available at the corresponding author with justifiable reason.

## Abbreviations

JBI: Jonna briggs institute
LLIN: Long lasting insecticidal nets
PRISMA: Preferred reporting items for systematic review and meta-analysis
