# Supplementary material for: Under utilization of long-lasting insecticidal nets (LLINs) is challenging malaria elimination program in Ethiopia: a systematic review and meta-analysis
Source: BMC Public Health. 2024 Mar 15;24:815. doi: 10.1186/s12889-024-18344-w (PMC10941431; doi:10.1186/s12889-024-18344-w)
Supplement: Supplementary file 1 — Supplementary Material 1. [file 12889_2024_18344_MOESM1_ESM.docx]

**Supplementary file: Some of the excluded studies and reasons for exclusion**

| **Authors** | **Region** | **study year** | **Study deign** | **study population** | **Sample Size** | **Response Rate (%)** | **Year of Publication** | **Number of outcomes** | **JBI Score (%)** | **Reason for exclusion** |
| --- | --- | --- | --- | --- | --- | --- | --- | --- | --- | --- |
| Zewde et al | Amhara, Oromia, Tigray, SNNPR | 2015 | Cross Sectional | House Holds | 1839 | 99.9 | 2017 | not reported | not assessed | outcome not measured |
| Sena et al | Oromia | 2012 | Comparative Cross Sectional | House Holds | 2,373 | 100 | 2013 | 1538 | not assessed | Outdated |
| Deressa | SNNP | 2008 | Cross Sectional | House Holds | 1230 | 100 | 2014 | 1906 | not assessed | Outdated |
| Woyessa et al | Oromia | 2008 | Cross Sectional | House Holds | 750 | 95 | 2014 | 142 | not assessed | Outdated |
| Gobena et al | Oromia | 2010 | Cross Sectional | House Holds | 2867 | 98.5 | 2012 | 630 | not assessed | Outdated |
| Hiruy et al | Amhara, Oromia, Tigray, SNNPR | 2015-2018 | Case-Control | House Holds | 5277 | _ | 2021 | 3510 | not assessed | inappropriate outcome measurement |
| Wubishet et al | Oromia | 2017 | Case–Control | Patients | 228 | _ | 2021 | 162 | not assessed | inappropriate outcome measurement |
| Biadgilign et al | Oromia | 2008 | Cross Sectional | House Holds | 335 |  | 2012 | 45 | not assessed | Outdated |
| Teklemariam et a | Harar | 2012 | Cross Sectional | House Holds | 784 | 97.3 | 2015 | 333 | not assessed | Outdated |
| Deressa et al | oromia and Amhara | 2009 | Cross Sectional | House Holds | 2874 | 100 | 2011 | 485 | not assessed | Outdated |
